# Supplementary material for: Unleashed Actin Assembly in Capping Protein-Deficient B16-F1 Cells Enables Identification of Multiple Factors Contributing to Filopodium Formation
Source: Cells. 2023 Mar 14;12(6):890. doi: 10.3390/cells12060890 (PMC10047565; doi:10.3390/cells12060890)
Supplement: Supplementary file 1 [file cells-12-00890-s001.zip › cells-2106261-supplementary/cells-2106261-supplementary 2nd revised back/SI and Movies/Supplementary Materials Hein et al revised.pdf]

Supplementary Materials:

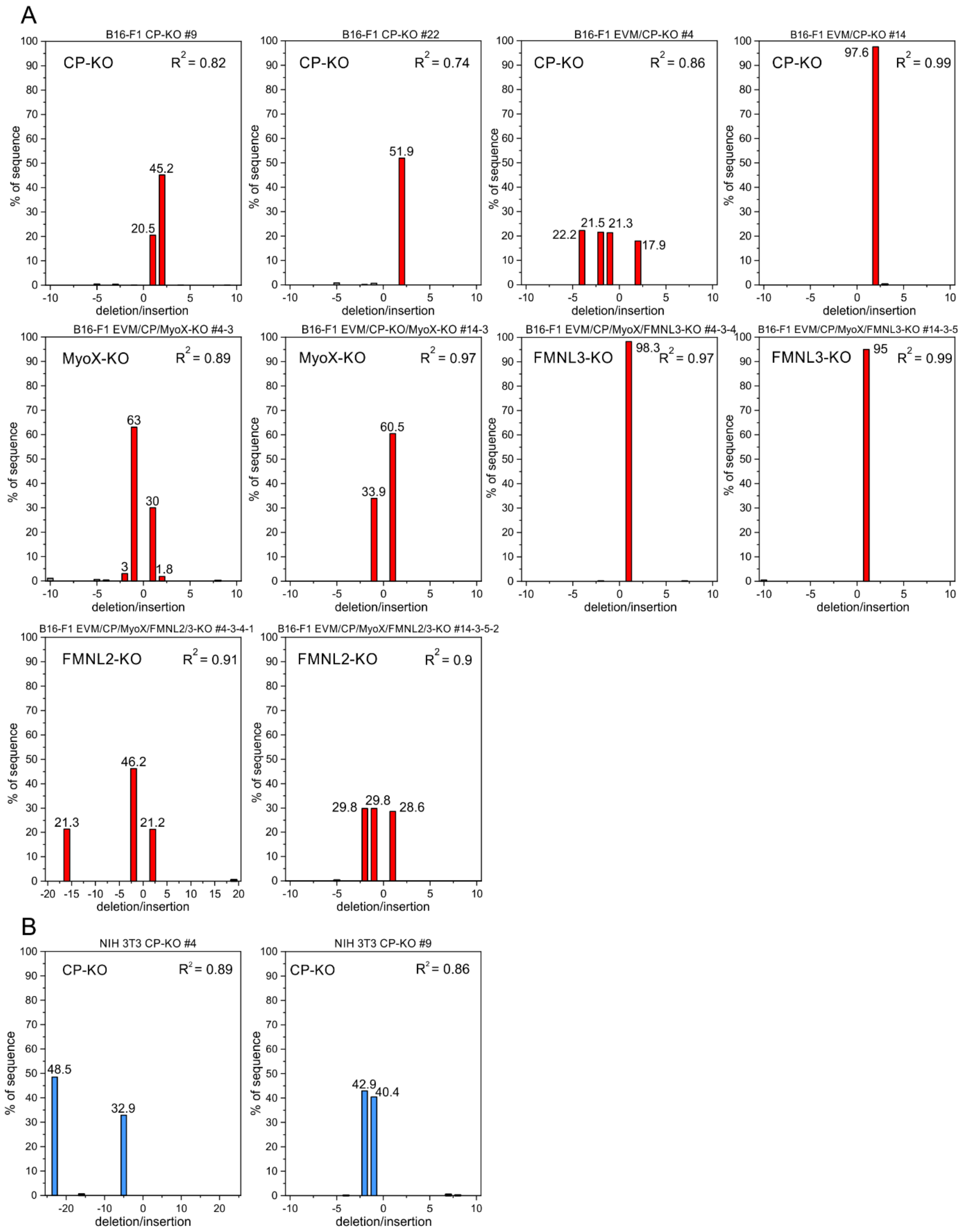

Figure S1. TIDE sequence trace decomposition analysis of clonal cell lines derived from B16-F1 cells (**A**) or NIH 3T3 fibroblast (**B**) used in this study. R^2 indicates goodness of fit. The B16-F1 derived CP-KO clone #9 carries an additional insertion of 179 bp and CP-KO clone #22 carries an additional insertion of 344 bp, respectively, as revealed by sequencing of the target sites. These large indels cannot be detected by TIDE analysis but also lead to frameshift mutations in the *Capzb* gene.

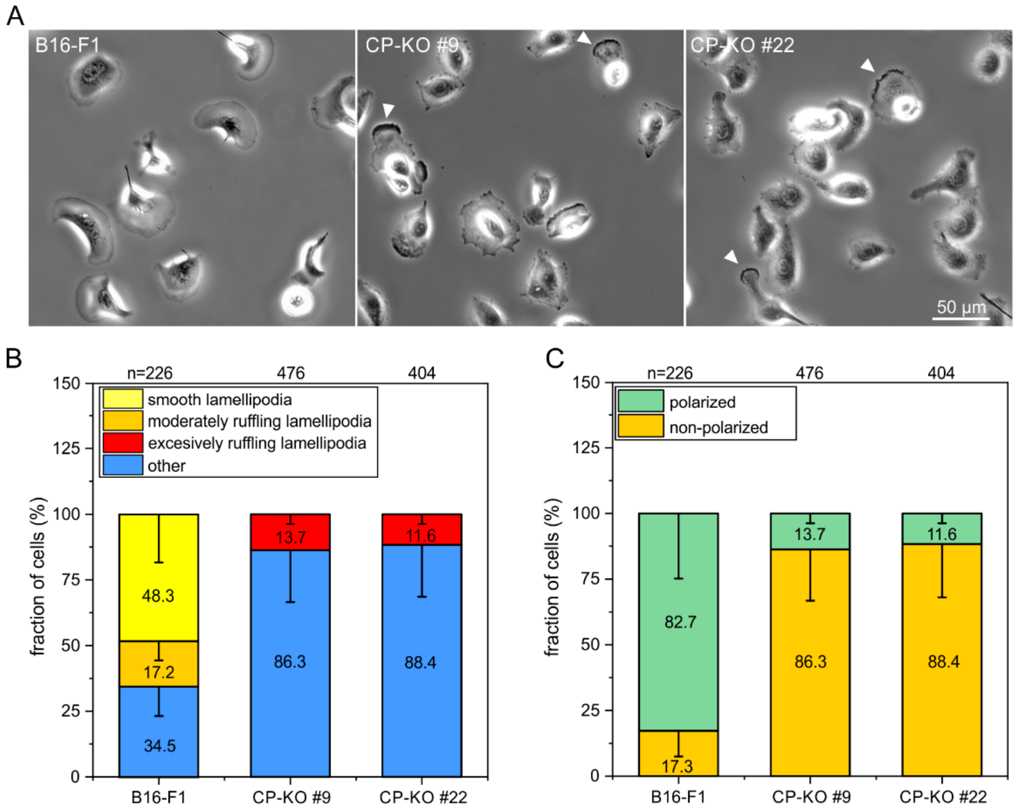

Figure S2. Loss of CP in B16-F1 cells prevents the formation of smooth lamellipodia and markedly decreases cell polarity. (**A**) Representative phase-contrast images of live B16-F1 control cells and derived CP-KO mutants on laminin. Excessively ruffling lamellipodia of CP-KO cells are highlighted by white arrowheads. (**B**) Quantification of cell morphology. (**C**) Quantification of cell polarity. Bars represent arithmetic means ± SD. n, number of cells analyzed from at least three independent experiments.

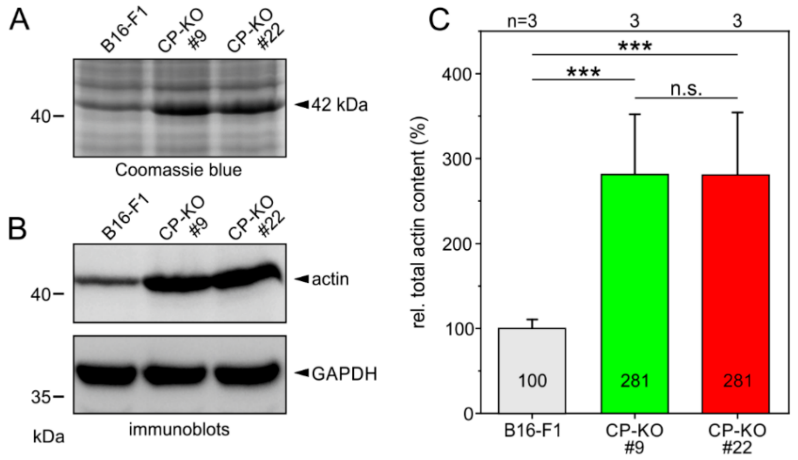

Figure S3. Loss of CP in B16-F1 cells increases global actin levels. (**A**) Proteins of total cellular lysates of B16-F1 cells and CP-KO mutant indicated, separated by SDS-PAGE and stained with Coomassie blue. (**B**) Corresponding immunoblot depicting global actin levels in B16-F1 and derived CP-KO mutants. Loading control: GAPDH. (**C**) Quantification of actin levels from immunoblots normalized to GAPDH expression as shown in (B). Bars represent arithmetic means ± SD. One-way ANOVA and Tukey Multiple Comparison test were used to reveal statistically significant differences between datasets. ***$p \leq 0.001$; n.s.; not significant. n, number of independent experiments.

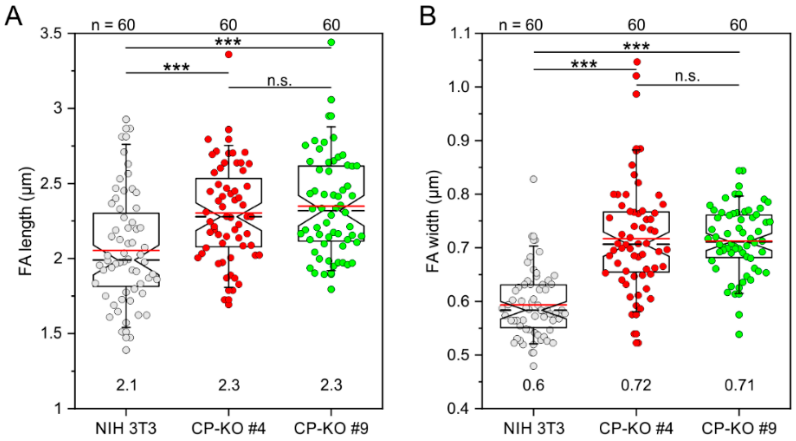

Figure S4. Loss of CP in NIH 3T3 fibroblasts increases length and width of FAs. (**A**) Quantification of FA length. (**B**) Quantification of FA width. The boxes in box plots indicate 50% (25-75%) and whiskers (5-95%) of all measurements, with dashed black lines depicting the medians, arithmetic means are highlighted in red. Non-parametric, Kruskal-Wallis test and Dunn's Multiple Comparison test were used to reveal statistically significant differences between datasets. ***p≤0.001; n.s.; not significant. n, number of cells analyzed from at least three independent experiments.

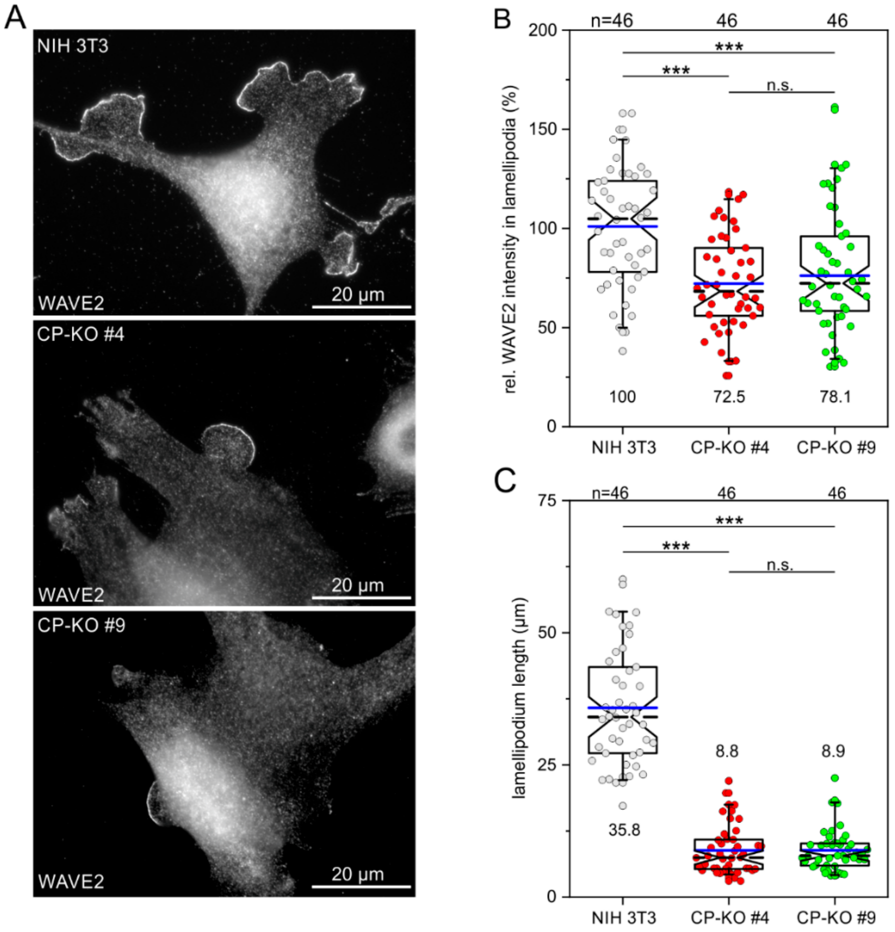

Figure S5. Loss of CP in NIH 3T3 fibroblasts suppresses lamellipodium formation. (**A**) Representative images of NIH 3T3 and derived CP-KO mutants cells migrating on fibronectin. Images display fixed cells stained for the lamellipodium marker protein WAVE2. (**B**) Quantification of WAVE2 intensity at the tips of lamellipodia. (**C**) Quantification of lamellipodium length. The boxes in box plots indicate 50% (25-75%) and whiskers (5-95%) of all measurements, with dashed black lines depicting the medians, arithmetic means are highlighted in blue. One-way ANOVA and Tukey Multiple Comparison test (B) and non-parametric, Kruskal-Wallis test and Dunn's Multiple Comparison test (C) were used to reveal statistically significant differences between datasets. ***$p \leq 0.001$; n.s.; not significant. n, number of cells analyzed from at least three independent experiments.

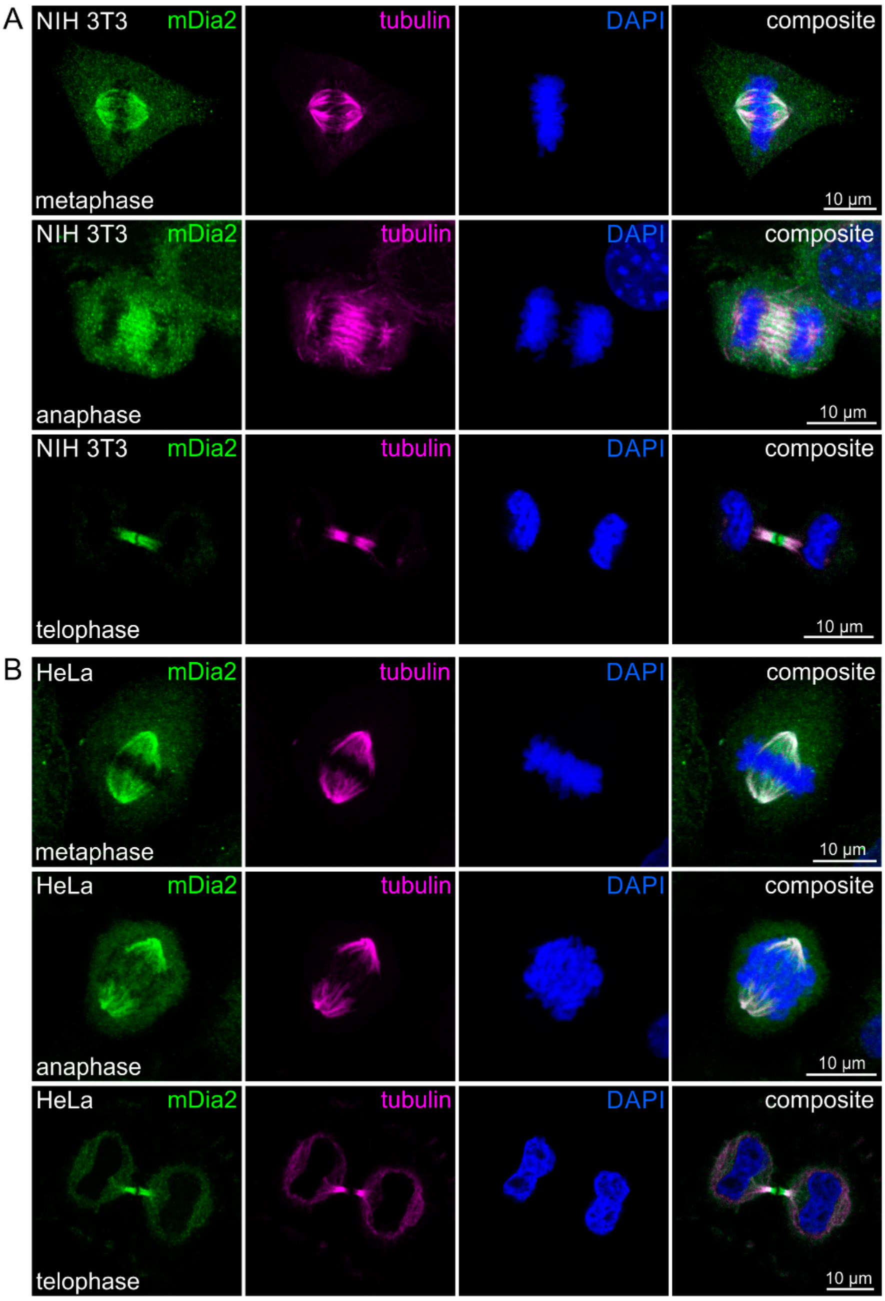

Figure S6. Endogenous mDia2 accumulates prominently together with tubulin at the mitotic spindle and midbody of dividing NIH 3T3 and HeLa cells. (**A**) Representative images of NIH 3T3 cells stained for endogenous mDia2 (green), tubulin (magenta) and DNA (blue) in metaphase (upper panel), anaphase (middle panel) and telophase (lower panel). (**B**) Representative images of HeLa cells stained for endogenous mDia2, tubulin and DNA in metaphase (upper panel), anaphase (middle panel) and telophase (lower panel).

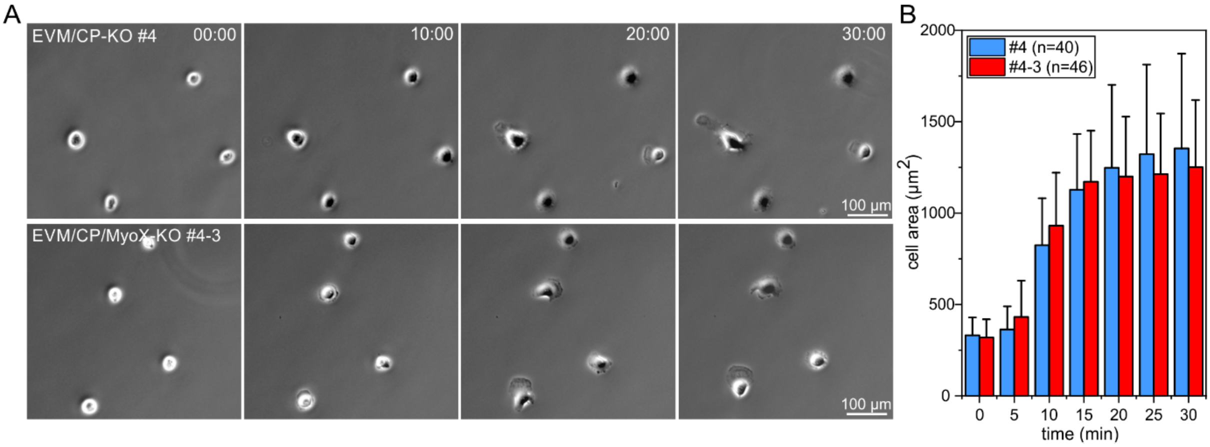

Figure S7. Loss of MyoX in EVM/CP cells does not noticeably affect cell spreading **(A)** Spreading of EVM/CP and EVM/CP/MyoX-KO mutant cells on laminin. Time is in min:sec. (B) Quantification of cell area over time. Data are means ± SD. Comparison of the two cell lines at the same time points by using Mann-Whitney U rank sum test did not reveal a statistical significance. n, number of analyzed cells from five independent experiments.

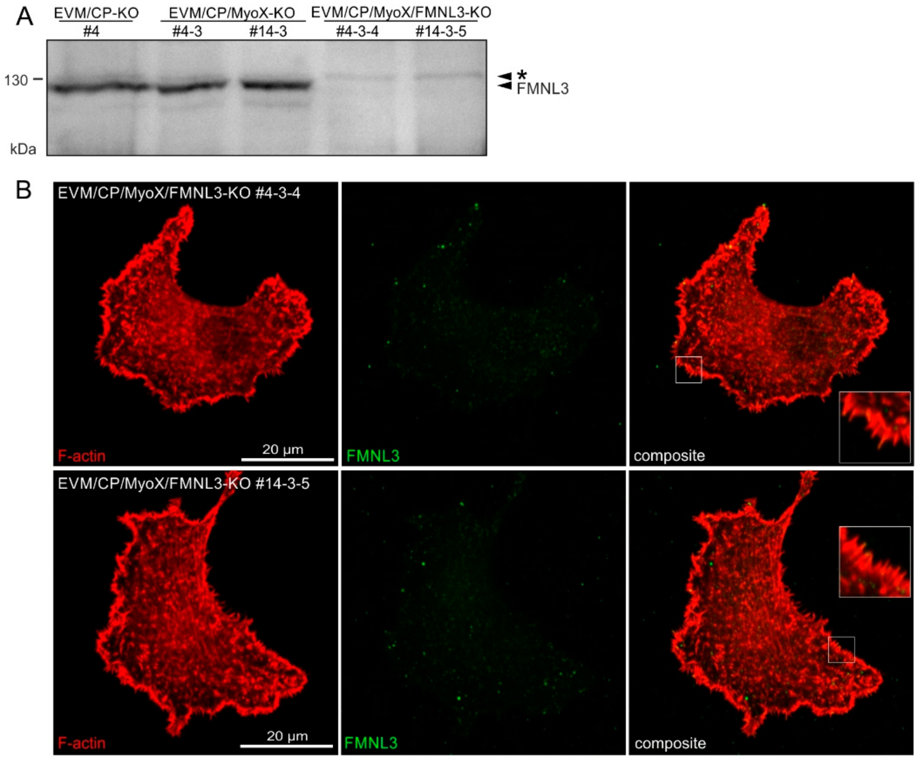

Figure S8. Additional evidence illustrating that the cross-reacting band shown in Figure 9A is nonspecific. (**A**) Improved resolution of proteins by extended run time in a 7.5% SDS-PAGE gel and subsequent immunoblotting of total proteins with FMNL3 antibodies allowed separation of the FMNL3-specific band from the non-specific band (indicated with an asterisk). (**B**) Loss of FMNL3 was additionally confirmed in independent EVM/CP/MyoX/FMNL3-KO mutant cells by immunofluorescence imaging, revealing the absence of FMNL3 at the tips of filopodia. Insets, enlarged images of boxed regions.

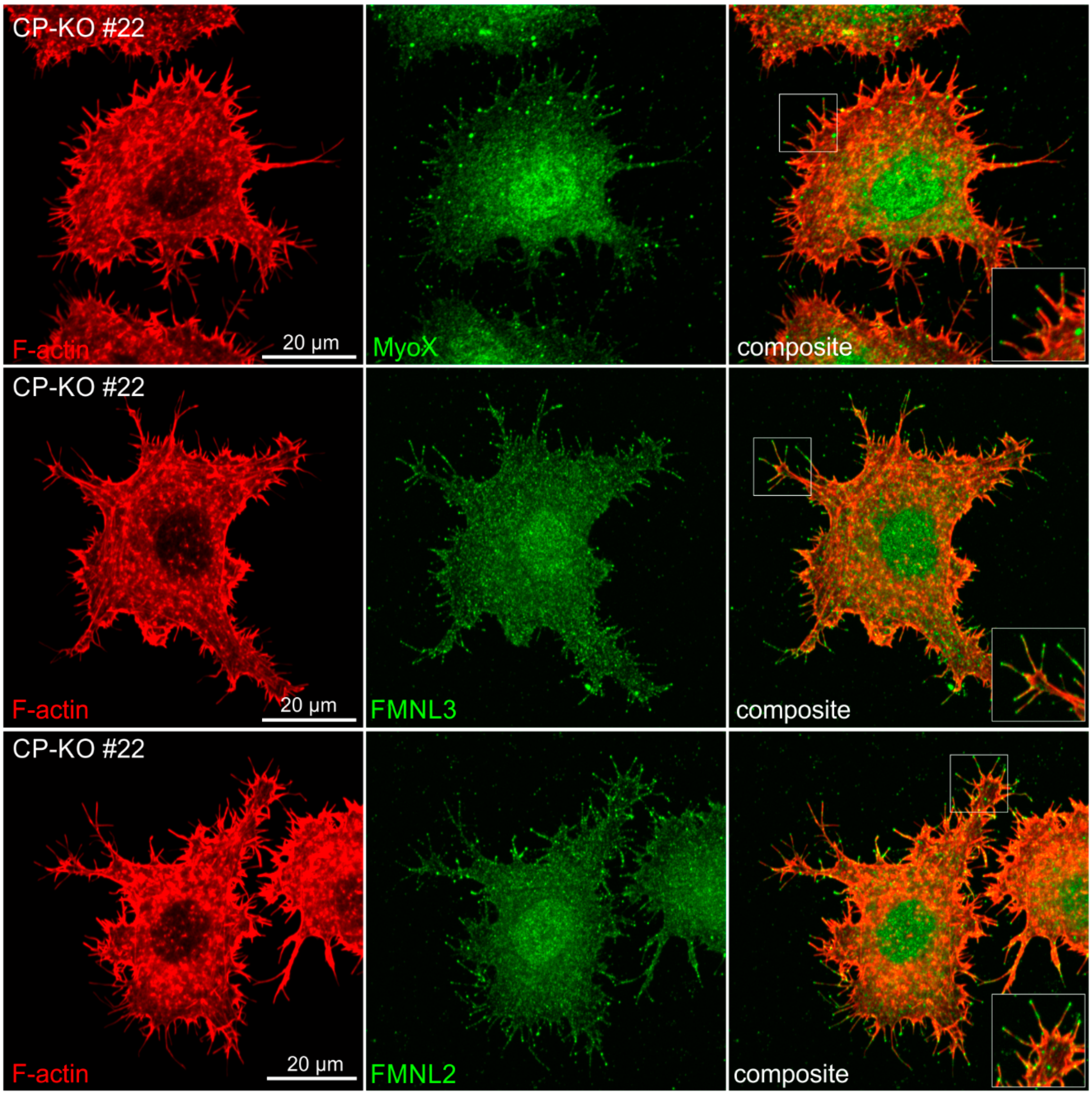

Figure S9. Endogenous MyoX, FMNL2 and FMNL3 are already present at the tips of filopodia in CP-KO cells. Representative CP-KO cells stained for endogenous MyoX and F-actin (upper panel), for endogenous FMNL2 and F-actin (middle panel), and for endogenous FMNL3 and F-actin (lower panel). Insets, enlarged images of boxed regions.

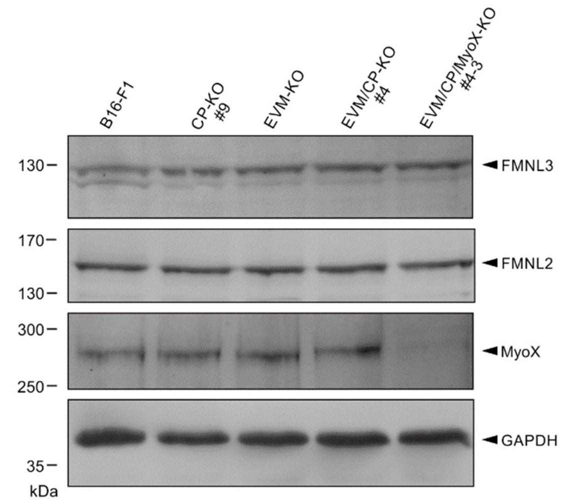

Figure S10. Global expression of FMNL3, FMNL2 and MyoX in representative clonal cell lines. Immunoblots of proteins as indicated in B16-F1 cells and one set of independent mutants. GAPDH was used as loading control. With the exception of EVM/CP/MyoX-KO mutant cells lacking MyoX, global expression of all tested proteins remained virtually unchanged.

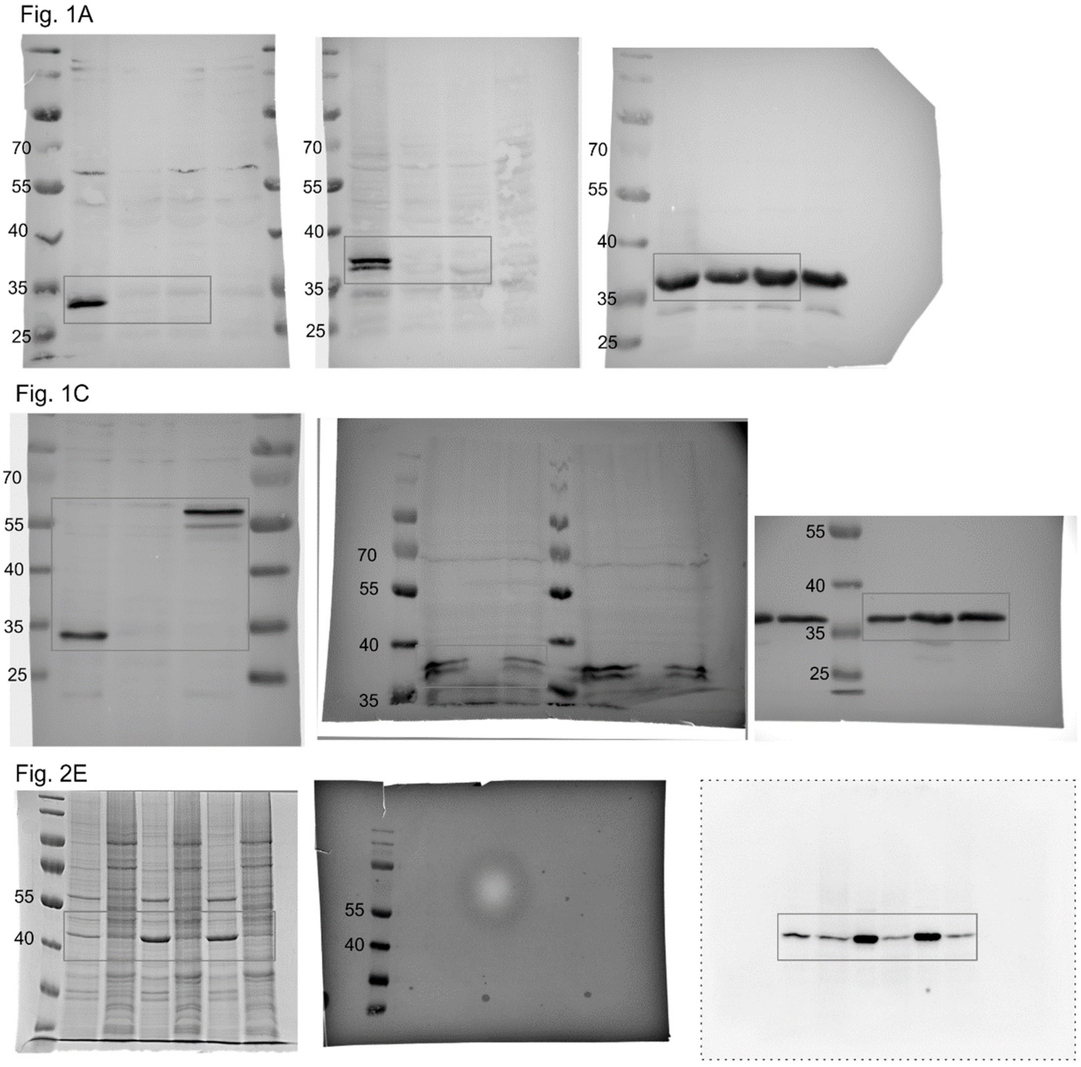

Fig. 3A

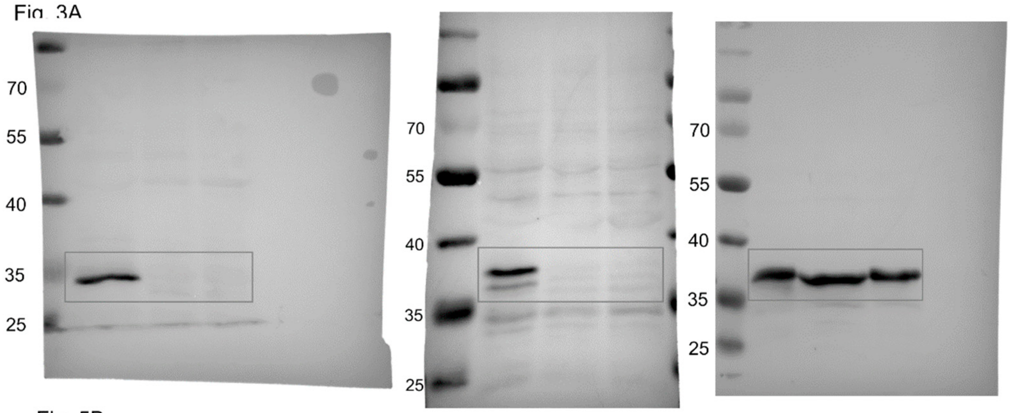

Fig. 5B

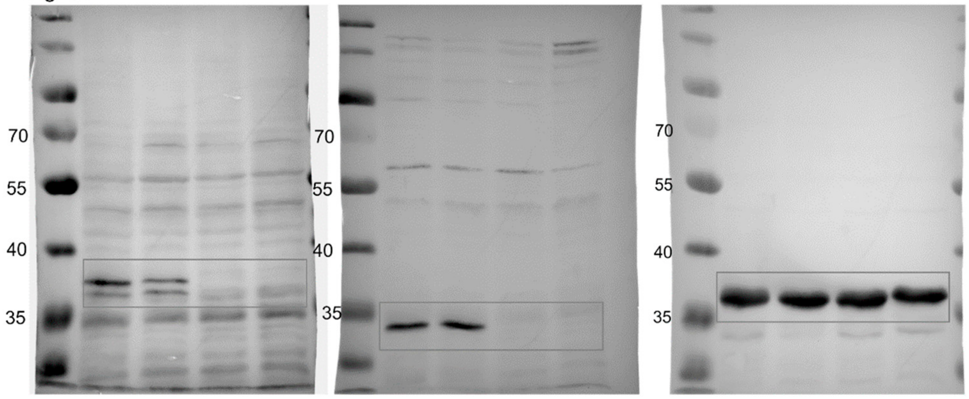

Fig. 8A

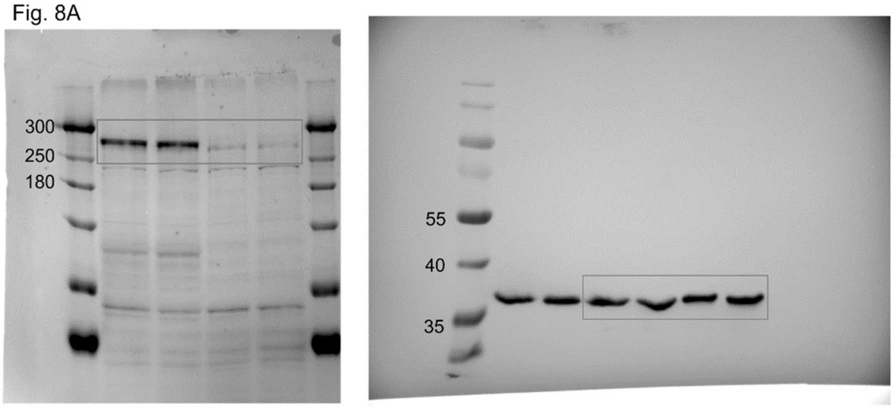

Fig. S9

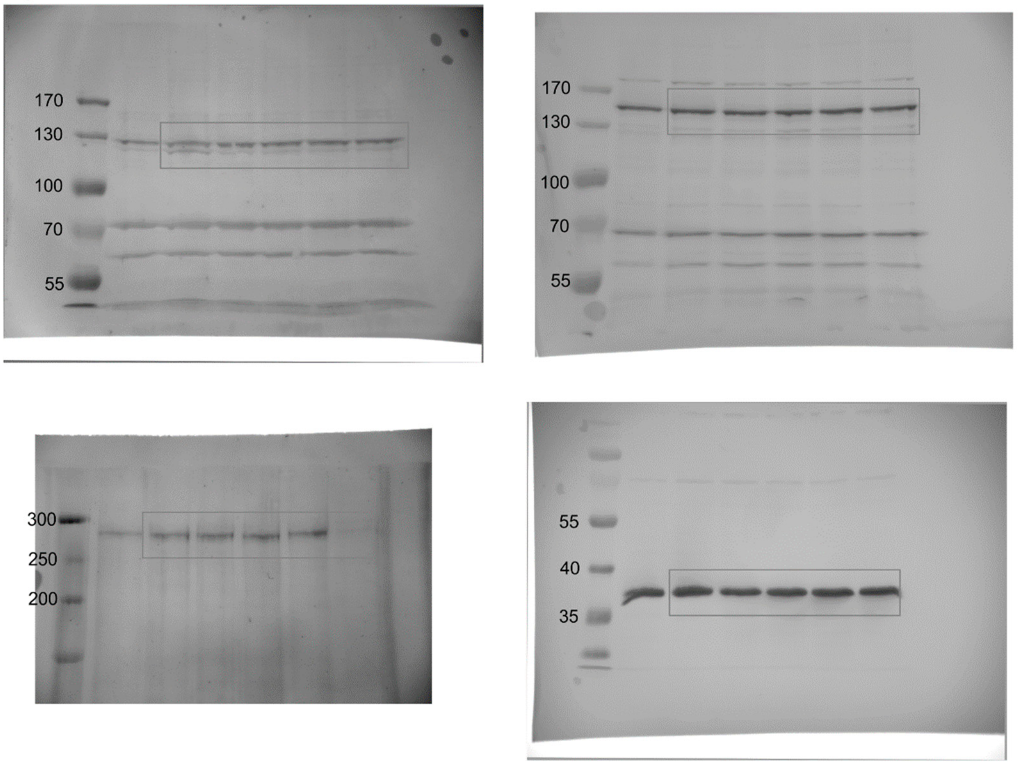

Figure S11. Uncropped immunoblots and Coomassie-stained gels. Boxes indicate regions that were used for main and supplemental figures.

Movie Legends:

Movie S1: Loss of CP in B16-F1 cells triggers the massive formation of filopodia. B16-F1 and CP-KO cells migrating on laminin were fixed and stained for F-actin with fluorescent phalloidin. Brightest point projection of 3D reconstructions from confocal Airyscan sections acquired with a 63x objective are shown. Scale bars, 20 µm.

Movie S2. Loss of CP in B16-F1 cells markedly perturbs lamellipodium dynamics and induces the formation of numerous filopodia. Random migration of B16-F1 cells and derived CP-KO mutants on laminin. Cells were recorded by phase-contrast time-lapse imaging using a 100x objective. Note considerably faster lamellipodium protrusion in B16-F1 wild-type cells (left) as compared with the CP-KO mutant forming excessively ruffling lamellipodia (middle). However, the majority of CP-KO cells were not polarized and instead formed numerous filopodia (right). Time is indicated in min:sec. Scale bar, 20 µm.

Movie S3: Filopodia in CP-deficient B16-F1 cells are highly dynamic. B16-F1 and CP-KO cells were transfected with EGFP-LifeAct to monitor actin dynamics during migration on laminin by time-lapse imaging using a 100x objective. As opposed to B16-F1 cells which developed smooth lamellipodia harboring embedded microspikes, the CP-KO mutant developed highly dynamics filopodia. Time is indicated in min:sec. Scale bar, 20 µm.

Movie S4. Loss of CP in B16-F1 cells severely impairs cell migration. Random migration of B16-F1 cells and derived CP-KO mutants on laminin. Cells were recorded by phase-contrast time-lapse imaging using a 4x objective with additional 1.6x optovar magnification and tracked by MTrackJ to illustrate representative cell trajectories. Note considerably faster movement of B16-F1 wild-type cells as compared with the CP-KO mutants. Time is indicated in h:min. Scale bar, 50 µm.

Movie S5. Loss of CP in NIH 3T3 fibroblasts also impairs cell migration. Random migration of NIH 3T3 fibroblasts and derived CP-KO mutants on fibronectin. Cells were recorded by phase-contrast time-lapse imaging using a 4x objective and tracked by MTrackJ to illustrate representative cell trajectories. Time is indicated in h:min. Scale bar, 100 µm.

Movie S6: B16-F1 cells lacking CP and all three Ena/VASP proteins still form prominent filopodia. EVM/CP-KO cells migrating on laminin were fixed and stained for F-actin with fluorescent phalloidin. Brightest point projection of 3D reconstructions from confocal Airyscan sections acquired with a 63x objective are shown. Scale bars, 20 µm.
